# Generation of motor neurons requires spatiotemporal coordination between retinoic acid and Mib-mediated Notch signaling

**DOI:** 10.1080/19768354.2018.1443494

**Published:** 2018-03-07

**Authors:** Hee Jeong Kong, Jae-Ho Ryu, Julan Kim, Ju-Won Kim, Bomi Seong, Ilson Whang, Jung Youn Park, Sang-Yeob Yeo

**Affiliations:** a Biotechnology Research Division, National Institute of Fisheries Science, Busan, Korea; b Department of Chemical and Biological Engineering, Hanbat National University, Daejeon, Korea; c Department of Genetic Resources Research, National Marine Biodiversity Institute of Korea (MABIK), Seochun, Korea

**Keywords:** Zebrafish, Notch, retinoic acid, motor neuron, mind bomb

## Abstract

Mind bomb (Mib) is an E3 ubiquitin ligase that activates the Notch signaling pathway. A previous study demonstrated that the generation of late-born GABAergic neurons may be regulated by the interplay between Mib and retinoic acid (RA). However, the relationship between Mib function and the retinoid pathway during the generation of late-born motor neurons remains unclear. We investigated the differentiation of neural progenitors into motor neurons by inhibition of Notch signaling and administration of RA to *Tg[hsp70-Mib:EGFP]* embryos. The number of motor neurons in the ventral spinal cord increased or decreased depending on the temporal inhibition of Mib-mediated Notch signaling. Inhibition of the retinoid pathway by citral treatment had a synergistic effect with overexpression of Mib:EGFP on the generation of ectopic motor neurons. Additionally, the proteolytic fragment of Mib was detected in differentiated P19 cells following treatment with RA. Our observations imply that the function of Mib may be attenuated by the retinoid pathway, and that Mib-mediated Notch signaling and the retinoid pathway play critical roles in the spatiotemporal differentiation of motor neurons.

## Introduction

In zebrafish embryos, primary neurogenesis gives rise to *elavr3*-positive differentiated primary motor neurons, interneurons and Rohon-Beard neurons, which populate the medial, intermediate and lateral domains on each side of the dorsal midline, respectively (Kim et al. 1996). This primary neurogenesis is regulated by a proneural gene, *ngn1*, and neurogenic gene, Notch, that limit the number of neuronal progenitors by lateral inhibition (Itoh et al. 2003). Genetics studies in zebrafish have identified *mind bomb* (*mib*) mutants, which are characterized by a severe neurogenic phenotype that has been interpreted as a deficit in Notch signaling (Itoh et al. 2003). The function of Mib as an E3 ubiquitin ligase is essential for efficient activation of Notch signaling in neighboring cells (Itoh et al. 2003). Our previous study showed not only that temporal overexpression of Mib:EGFP caused an increase in the number of GABAergic Kolmer-Adduhr (KA) cells in the p3 domain of the ventral spinal cord, but also that these phenotypes were suppressed by exogenous retinoic acid (RA) (Ryu et al. 2015). RA is a vitamin A metabolite that acts upstream of prepatterning genes that regulate the expression of proneural genes, and it specifies the identity of motor neurons in the pMN domain of the spinal cord (Sockanathan and Jessell 1998; Franco et al. 1999). However, the relationship between Mib function and the retinoid pathway during generation of motor neuron at neurula stage remains unclear. Here we investigated spinal motor neuron differentiation by inhibition of Mib-mediated Notch signaling and administration of RA or citral to *Tg*[*hsp70-Mib:EGFP*] embryos. We also assessed the effect of Mib:EGFP overexpression on the generation of motor neurons during primary neurogenesis. To determine whether the retinoid pathway modulates Mib directly or indirectly, we examined the stability of Mib using in P19 cells. Mouse P19 embryonic carcinoma cells are uncommitted, multipotent cells that can be differentiated into neuronal and glial cells following RA treatment (Jones-Villeneuve et al. 1982). We compared the stability of Mib in uncommitted P19 cells to that in committed P19 cells. Our observations imply that the function of Mib may be attenuated by the retinoid pathway, and that Mib-mediated Notch signaling and the retinoid pathway play critical roles in the spatiotemporal differentiation of motor neurons.

## Materials and methods

### Fish lines and mutants

Zebrafish were maintained as described by Kong et al. (2015). AB* wild-type and *Tg*[*hsp70-mib:EGFP*] fish (Ryu et al. 2015) were used for all experiments.

### Plasmids

A plasmid used for *in vivo* expression were generated by subcloning either polymerase chain reaction (PCR)-amplified or restriction enzyme-digested fragments of *mindbomb* into the pCS3 + MT vector. Flag-tagged Mib were transfected into P19 cells.

### Preparation of RA

All-trans RA (Sigma-Aldrich) was prepared as a stock solution at 10^−2 ^M in 95% ethanol (Jones-Villeneuve et al. 1982). The stock solution was added directly to the culture medium or 30% Danio solution to dilute to the desired concentration (1 × 10^−7 ^M and 5 × 10^−7 ^M for P19 cells and zebrafish embryos, respectively).

### P19 cell culture, transfection and Western blot analysis

The embryonal carcinoma cell line, P19 (ATCC CRL-1825), was grown in alpha medium (Invitrogen) supplemented with 2.5% fetal calf serum and 7.5% calf serum (Invitrogen). All cultures were maintained at 37°C in a 5% CO_2_ atmosphere. P19 cell differentiation was carried out as follows: cells were dissociated using 0.25% trypsin and 1 mM EDTA (trypsin/EDTA) and plated at a density of 2 × 10^5 ^cells/mL into a bacteriological-grade Petri dish, where they aggregated spontaneously in the presence of RA. The medium was replaced after 2 days. The aggregates were dissociated using trypsin/EDTA and plated into new tissue culture dishes. These cells were then used for transfection the following day.

P19 cells were transiently transfected with 1.5 µg of each plasmid DNA per 6-cm dish using FuGENE®6 (Roche). Transfected cells were treated 9 h later with MG132 at a final concentration of 0.1 µM overnight. On the following day, cells were harvested and lysed in lysis buffer (20 mM HEPES [pH 7.5], 150 mM KCl, 0.5% NP-40 and 10% glycerol) containing a protease inhibitor cocktail tablet (Roche). Proteins were electrophoresed on a 4–12% NuPAGE gel and transferred to a polyvinylidene difluoride membrane (Invitrogen), which was subsequently incubated with a 1:5000 diluted anti-FLAG M2 (Sigma-Aldrich) overnight at 4°C. The signal was then visualized using a horseradish peroxidase-conjugated secondary antibody (anti-mouse, anti-rabbit or anti-rat, all at a 1:5000 dilution, Santa Cruz Biotechnology) and a chemiluminescence detection system (Pierce).

### Whole-mount *in situ* hybridization

Whole-mount *in situ* hybridization was performed as described by Kong et al. (2015). Anti-sense riboprobes were transcribed from zebrafish cDNAs encoding *ngn1* (Kim et al. 1997), *her4* (Jung et al. 2012), *islet1* (Inoue et al. 1994) and *elavr3* (Kim et al. 1996). Images were taken using a differential interference contrast microscope (Axioplan2, Carl Zeiss).

## Results and discussion

### Inhibtion of Notch signaling by overexpression of Mib:EGFP

**Figure 1.**
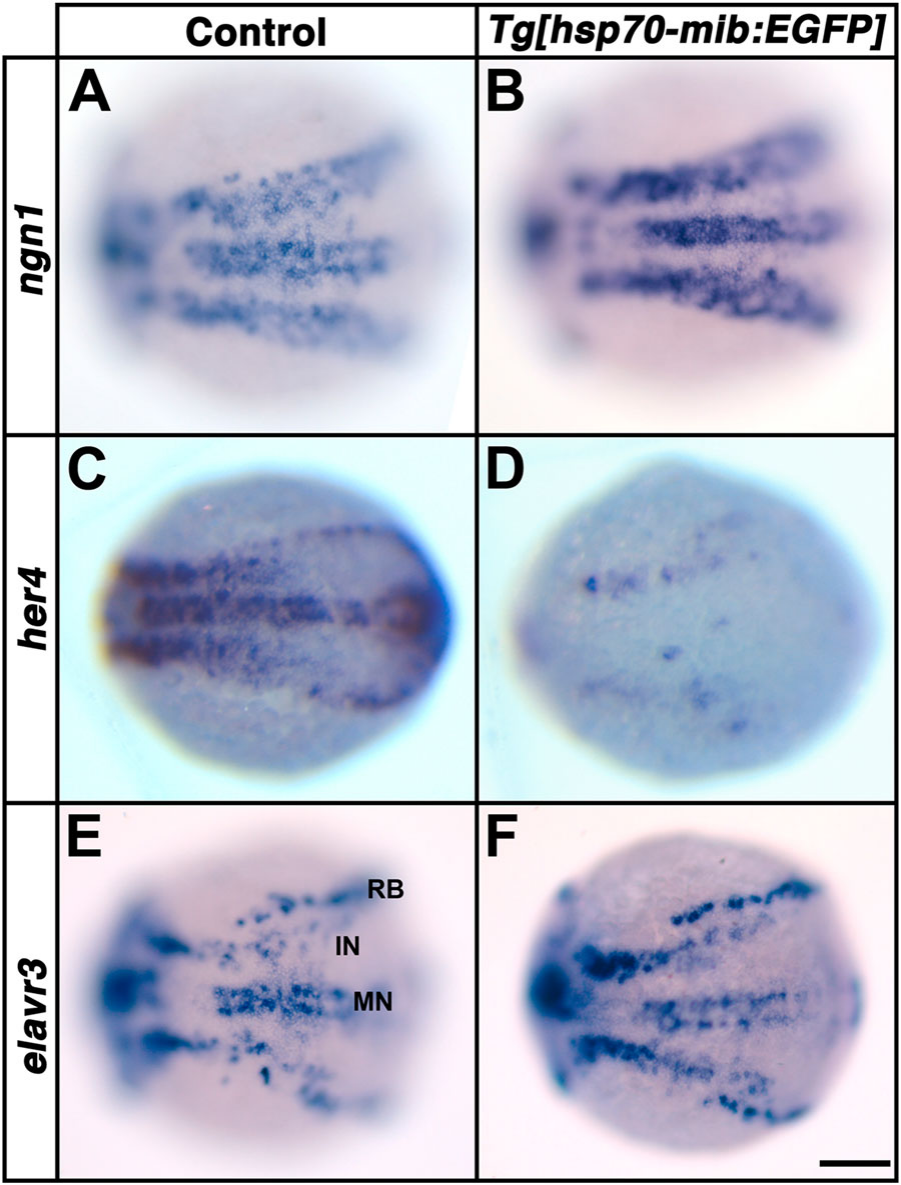
Overexpression of Mib:EGFP causes a reduction in Notch signaling. Dorsal views. Anterior to the left. The expression of *ngn1* (A, B), *her4* (C, D) and *elarvr3* (E, F) in control (A, C and E) and heterozygous *Tg[hsp70-mib:EGFP]* embryos at 11 hpf (B, D and F) following heat-shock at 6 hpf. RB, Rohon-Beard neuron; IN, interneuron; MN, motor neuron. Scale bar: 100 µm.

We previously observed the neurogenic phenotype of *Tg*[*hsp70-mib:EGFP*] embryos after gastrulation (13 h after post-fertilization, hpf) by heat-shocking during the early gastrulation stage (7.5 hpf) (Ryu et al. 2015). Our observations implicated that overexpression of Mib:EGFP has a lesser effect on the generation of ectopic motor neurons than those of sensory neurons and interneurons during late gastrulation (Ryu et al. 2015). However, the primary neurogenesis starts at 8.5 hpf known as a late-gastrulation stage by expressing *ngn1* as a proneural gene, and *elavr3* as a neuronal marker is first expressed at 11 hpf in zebrafish embryos (Kim et al. 1997). Experimentally, there was a two-hour time-delay to express an ectopic EGFP fuzed protein after heat-shocking a transgenic zebrafish under control of heat-shock 70 promoter (Yeo et al. 2001). Thus, it is unclear whether the neurogenic phenotype can be observed within the motor neuron domain of *Tg*[*hsp70-mib:EGFP*] embryos heat-shocked before gastrulation (5.5 hpf), and whether overexpression of Mib:EGFP effectively inhibits the Notch signaling pathway. To determine the effects of Mib:EGFP overexpression on the primary neurogenesis of motor neurons, we performed whole-mount *in situ* hybridization using *ngn1*, *her4* and *elavr3* riboprobes in wild-type and *Tg*[*hsp70-mib:EGFP*] embryos heat-shocked at 5.5 hpf (Figure 1).

Beginning at mid-gastrulation, zebrafish *ngn1* mRNA was expressed in the proneural clusters in a salt-and-pepper manner (Kim et al. 1997, Figure 1(A)), whereas far denser *ngn1*-expressing cells were observed in *Tg*[*hsp70-mib:EGFP*] embryos heat-shocked before gastrulation (Figure 1(B)). These results are consistent with the observation that a proneural gene is negatively regulated within the proneural domain by a lateral inhibition mechanism. Our observation also implies that the Notch signaling pathway is impaired by overexpression of Mib:EGFP. To test this hypothesis, we performed *in situ* hybridization to evaluate expression of *her4*, a direct target of Notch-mediated signaling (Yeo et al. 2007; Jung et al. 2012). At the tailbud stage, two distinct stripes of *her4* expression appeared laterally: intermediate and slightly medial to the other (lateral) (Yeo et al. 2007, data not shown). The lateral and medial stripes extended further caudally and surrounded the tailbud in wild-type embryos at the 1-somite stage (Figure 1(C)). However, Mib:EGFP-overexpressing embryos exhibited a dramatic reduction in *her4* expression (Figure 1(D)). To understand the effect of Mib:EGFP expression better, we monitored the expression of *elavr3*, a pan-neuronal marker (Kim et al. 1996, Figure 1(E)). Rohon-Beard sensory neurons, interneurons and motor neurons are generated in the lateral, intermediate and medial stripes, respectively (Kim et al. 1996). The number of *elavr3*-expressing cells was increased in the lateral and intermediate stripes, but not in the medial stripes (Figure 1(F)). The neurogenic phenotype was observed in the lateral and intermediate stripes of *Tg*[*hsp70-mib:EGFP*] embryos heat-shocked before gastrulation. Although temporal overexpression of Mib:EGFP effectively inhibited Notch signaling, it had a lesser effect on the generation of motor neurons than those of sensory neurons and interneurons, even when Notch signaling was inhibited during early gastrulation by Mib:EGFP overexpression. These observations implicate a factor and/or signaling pathway that counterbalances the effect of Mib:EGFP overexpression on generation of primary motor neurons during early gastrulation.

### Specification of neurons in the ventral spinal cord at the early neurula stage

**Figure 2.**
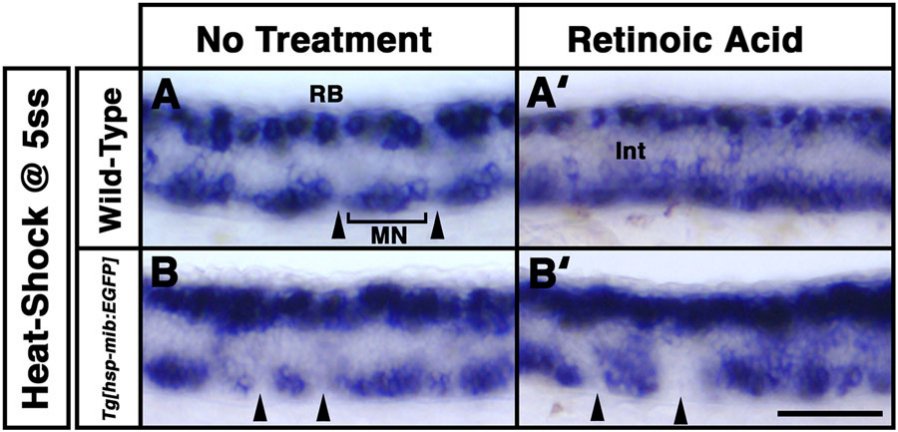
Overexpression of Mib:EGFP attenuates the effect of exogenous retinoic acid. Lateral views. Anterior to the left. At 26 hpf, expression of *islet1* between the 8th and 11th somites of embryos following no treatment (A and B) and retinoic acid (RA) treatment (A’ and B’). Wild-type (A and A’) and *Tg[hsp70-mib:EGFP]* (B and B’) embryos were heat-shocked at the 5-somite stage (ss). Motor neuron (MN) clusters (brackets) located in a somite flanking a non-MN domains (arrowhead) in wild-type and *Tg[hsp70-mib:EGFP]* embryos. Scale bar: 100 µm.

Our previous study suggested that RA signaling attenuates the effect of Mib:EGFP overexpression on the generation of GABAergic neurons (Ryu et al. 2015). This observation suggests the idea that endogenous RA might act non-cell autonomously to bias proliferating neural progenitors to differentiation into different types of neurons rather than GABAergic interneurons. The retinoid-induced increase in the number of progenitor cells appears to result in an increase in that of motor neurons in the embryonic chick spinal cord (Sockanathan and Jessell 1998). To test this hypothesis at the early neurula stage, we performed *in situ* hybridization to evaluate *islet1* expression in wild-type and *Tg*[*hsp70-mib:EGFP*] embryos treated with RA after heat-shock at the 5-somite stage (Inoue et al. 1994, Figure 2). At 26 hpf, motor neurons are composed of units called motor neuron clusters within each somite, and these somatic motor neurons control body muscles in the ventral spinal cord (Figure 2(A)). In wild-type embryos treated with RA at the 5-somite stage, the number of motor neurons dramatically increased in the ventral spinal cord including the non-motor neuron domain franking motor neuron clusters at 26 hpf (Figure 2(A’)). In contrast to our observation, RA treatment at the 5-somite stage causes a decrease in the number of GABAergic neurons (Ryu et al. 2015). These observations were consistent with the idea that endogenous RA might act as a non-cell autonomously bias on proliferating neural progenitors to differentiation into motor neurons rather than GABAergic interneurons in the ventral spinal cord. In *Tg*[*hsp70-mib:EGFP*] embryos heat-shocked at the 5-somite stage, the overall number of motor neurons remained unchanged or reduced in some motor neuron clusters, whereas that of sensory neurons was increased (Figure 2(B)). In *Tg*[*hsp70-mib:EGFP*] embryos treated with RA after heat-shock at the 5-somite stage, the number of interneurons and motor neurons increased in the motor neuron cluster of ventral spinal cord, but not in the non-motor neuron domain franking motor neuron clusters at 26 hpf (Figure 2(B’)). In contrast to our observation, *Tg*[*hsp70-mib:EGFP*] embryos treated with RA after heat-shock at the 5-somite stage showed an increase in the number of GABAergic neurons in the ventral spinal cord (Ryu et al. 2015). Taken together with our observations, these studies suggests that Mib-mediated Notch signaling pathway might act cell autonomously to bias proliferating neural progenitors to differentiation into GABAergic interneurons rather than motor neurons in the ventral spinal cord at the early neurula stage.

### Specification of neurons in the ventral spinal cord at the mid-neurula stage

**Figure 3.**
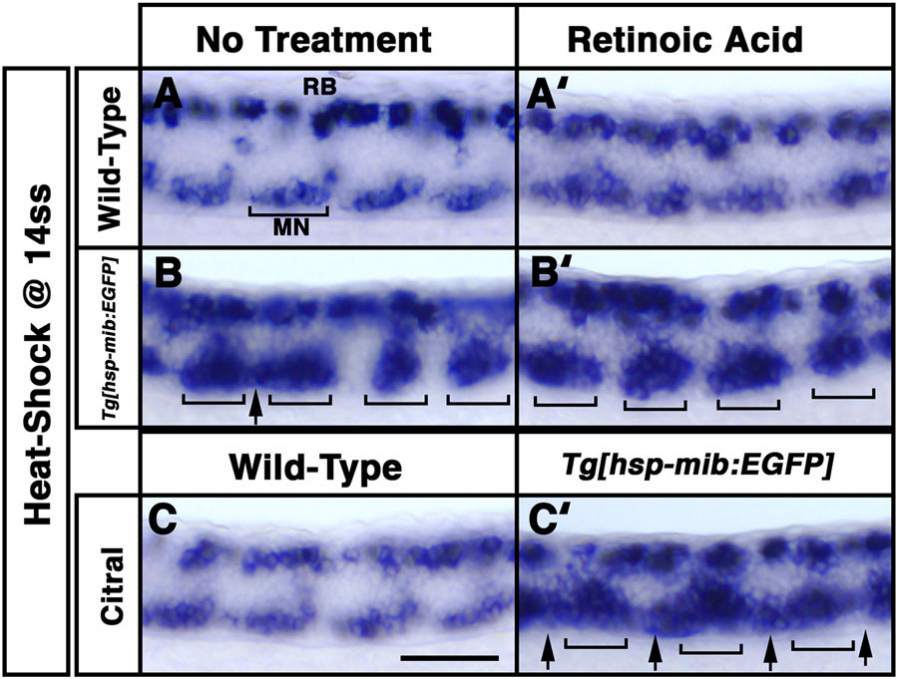
Retinoid pathway attenuates the effect of overexpression of Mib:EGFP. Lateral views. Anterior to the left. At 26 hpf, expression of *islet1* between the 8th and 11th somites of embryos following no treatment (A–C), retinoic acid (RA) treatment (A’ and B’) or citral treatment (C and C’). Wild-type (A, A’ and C) and *Tg[hsp70-mib:EGFP]* (B, B’ and C’) embryos were heat-shocked at the 14-somite stage (ss). Motor neuron (MN) clusters (brackets) located in the somites flanking non-MN domain in wild-type embryos. Ectopic motor neurons (arrows) filled the gaps between motor neuron clusters (brackets) in *Tg[hsp70-mib:EGFP]* embryos following heat-shock at the 14ss (B) and exposure to citral at the 14ss (C’). Scale bar: 100 µm.

When the neural progenitors are competent to differentiate as a specific subtype of neurons, Notch signaling limits the number of cells that adopt this cell fate (Yeo and Chitnis 2007). For example, inhibition of Notch signaling by overexpression of XdnSu(H)myc at 7 hpf (primary neurogenesis stage) reduced the number of GABAergic KA cells, whereas heat shock between 10 and 14 hpf (early neurula stage) increased the number of GABAergic neurons, and heat shock at 17 hpf had little effect on the number of GABAergic neurons in zebrafish embryos (Yeo and Chitnis 2007). To assess the relationship between retinoid pathway and Mib-mediated Notch signaling in the generation of motor neurons at the mid-neurula stage, we performed *in situ* hybridization to evaluate *islet1* expression in wild-type and *Tg*[*hsp70-mib:EGFP*] embryos treated with RA after heat-shock at the 14-somite stage (Figure 3). In wild-type embryos treated with RA at the 14-somite stage, some ectopic motor neurons were observed in the motor neuron clusters of the ventral spinal cord at 26 hpf (Figure 3(A’)). In *Tg*[*hsp70-mib:EGFP*] embryos heat-shocked at the 14-somite stage, the number of motor neurons dramatically increased within motor neuron clusters (Figure 3(B)). These observations are consistent with a role for the retinoid pathway in promoting motor neuron differentiation (Figure 3(A’)) and suggest that the generation of late-born motor neurons requires temporal regulation of the Mib-mediated Notch signaling pathway (Figure 3(B)). Additionally, some ectopic motor neurons were observed in the non-motor neuron domain located between motor neuron clusters in *Tg*[*hsp70-mib:EGFP*] embryos heat-shocked at the 14-somite stage (Figure 3(B)), but not in the presence of RA (Figure 3(B’)). Endogenous RA and *mib* are highly expressed in the somite and spinal cord at the 14-somite stage, respectively, raising the possibility that the function of Mib may be attenuated by the retinoid pathway. To test this hypothesis, embryos were exposed to citral, an inhibitor of RA formation from retinol (Connor and Smit 1987). Motor neurons were located on motor neuron clusters flanked laterally by non-motor neuron domains in wild-type and *Tg*[*hsp70-mib:EGFP*] embryos (Figure 3(A–3C)). At 26 hpf, the number of motor neurons dramatically increased in the ventral spinal cord of *Tg*[*hsp70-mib:EGFP*] embryos treated with citral after heat-shock at the 14-somite stage (Figure 3(C’)). These results suggested that citral treatment and overexpression of Mib:EGFP had synergistic effects on the generation of ectopic motor neurons, and implying that the retinoid pathway attenuated the Mib-mediated Notch signaling pathway. When the neural progenitors are competent to differentiate at 5-somite stage, Mib-mediated Notch signaling might limit the number of cells that adopt the GABAergic neurons as a cell fate (Ryu et al. 2015). However, when the neural progenitors in the non-motor neuron domain maintain their multipotency at 14-somite, they might require the Mib-mediated Notch signaling as well as retinoic pathway. Although our observations implying that the retinoid pathway attenuated the Mib-mediated Notch signaling pathway, it is unclear whether the retinoid pathway modulates the function of Mib directly or indirectly.

### Retinoid pathway promotes the proteolysis of Mib

**Figure 4.**
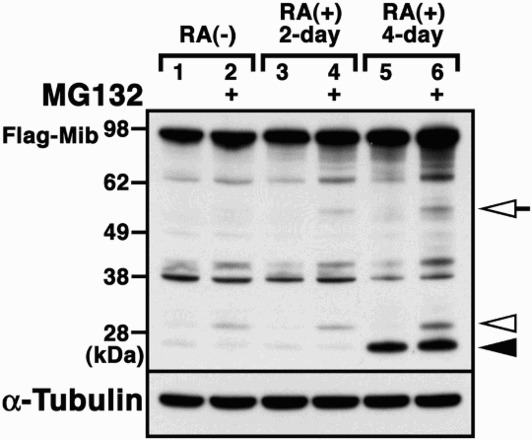
Proteolysis of Mib in the presence of retinoic acid. Flag-tagged Mib (Flag-Mib) was transfected into P19 cells (lanes 1 and 2), P19 cells treated with retinoic acid (RA) for 2 days (lanes 3 and 4) or P19 cells treated with RA for 4 days (lanes 5 and 6) in the absence or presence of MG132. Western blot analysis using the anti-Flag antibody revealed the proteolytic fragments of Mib. Arrowhead and open arrowhead indicates the retinoid pathway-dependent 27 kDa fragments and proteasome-dependent 30 kDa fragments, respectively. Open arrows indicate the proteolytic fragments, depending on both the retinoid pathway- and proteasome-dependent 55 kDa fragments.

We next examined the function of Mib in P19 cells, which differentiate into neurons following RA treatment (Jones-Villeneuve et al. 1982). We found Mib proteolysis during the ubiquitination assay in differentiated P19 cells by RA (data not shown). To assess the effect of RA on the stability of Mib, the N-terminal Flag-tagged Mib plasmid was transfected into P19 cells aggregated for 2 and 4 days in the presence of RA and/or MG132, which effectively blocked the proteolytic activity of the 26S proteasome complex (Jones-Villeneuve et al. 1982; Tsubuki et al. 1993). Western blot analysis was performed using an anti-Flag antibody of whole cell lysates from P19 and differentiated P19 cells transiently transfected with the Flag-tagged Mib (Figure 4). Although it was difficult to confirm the proteasome-dependent degradation of full-length Mib in P19 cells transfected with Flag-tagged Mib alone (lane 2 in Figure 4), we observed 55-kDa, 30-kDa and 27-kDa fragments of Mib (open arrow, open arrowhead and arrowhead in Figure 4, respectively). In the presence of MG132, 55-kDa and 30-kDa fragments of Mib were detected (lanes 2, 4 and 6 in Figure 4); thus, both fragments were generated in a proteasome-dependent manner (Figure 4). The 55-kDa fragment of Mib was observed in the presence of MG132 in differentiated P19 cells treated with RA (lanes 4 and 6 in Figure 4), whereas the 27-kDa fragment was detected even in the absence of MG132 (lane 5 in Figure 4). This observation implies that Mib may undergo proteolytic cleavage in differentiated P19 cells treated with RA as a proteasome-independent manner, suggesting that the retinoid pathway might attenuate the Mib-mediated Notch signaling pathway by promoting proteolysis of Mib.

Temporal overexpression of Mib:EGFP exhibits unique features. First, its effect on primary neurogenesis of motor neurons may be attenuated, although it effectively suppresses Notch signaling (Ryu et al. 2015). Second, overexpression of Mib:EGFP causes an increase or decrease in the number of motor neurons, depending on temporal inhibition of the Mib-mediated Notch signaling pathway. Finally, exogenous RA suppresses the effect of Mib:EGFP overexpression on the generation of ectopic motor neurons between motor neuron clusters. Additionally, inhibition of the retinoid pathway by treatment of citral has a synergistic effect on the generation of ectopic motor neurons by overexpression of Mib:EGFP. In this study, we showed not only that the function of Mib may be attenuated by retinoid pathway *in vivo*, but also that a protease may induce proteolysis of Mib in differentiated P19 cells following treatment with RA.
